# British Association of Sexual Health and HIV UK national guideline for the management of anogenital herpes, 2024

**DOI:** 10.1177/09564624241282396

**Published:** 2024-10-07

**Authors:** Raj Patel, John Green, Benjamin Moran, Emily Clarke, Kanchana Seneviratne, Ceri Evans, Felicity Young, Marian Nicholson, Emanuela Pelosi, Margaret Kingston, Elizabeth Foley

**Affiliations:** 1 Royal South Hants Hospital, Southampton, UK; 2 543413 Smith and Rowcroft Draughting Ltd, St Mary’s Hospital, London, UK; 3 156767 Croydon University Hospital, London, UK; 4 4595 Liverpool University Hospital NHS Foundation Trust, Liverpool, UK; 5 7852 Somerset NHS Foundation Trust, Taunton, UK; 6 112004 Chelsea and Westminster Hospital, London, UK; 7 232267 Solent Sexual Health, Isle of Wight, UK; 8 Herpes Viruses Association, London, UK; 9 105634 University Hospitals Trust, Southampton, UK; 10 5293 Manchetser University NHS Trust, Manchester, UK

**Keywords:** herpes, antiviral, Europe, herpes simplex virus

## Abstract

The guideline provides recommendations on the management of adults with anogenital herpes in the UK. Recommendations include diagnostic tests, management of the primary or first episode of anogenital herpes and recurrences, effectiveness of therapy, prophylaxis, and prevention of transmission between partners, as well as patient centred counselling.

## What’s new in the 2024 guidelines?

• A section on herpes proctitis.
• An updated box on HSV transmission: Key points to cover with patients.
• Removal of detailed advice on the management of HSV in pregnancy – this now has a separate joint BASHH/RCOG guideline.
• An expansion of the science and utility of serological testing
• Updated prices for antivirals

## Introduction and methodology

### Objectives

The overall aim of the guideline is to prevent morbidity (physical and psychological) associated with genital herpes and ultimately to reduce transmission and prevalence. For some groups, particularly those at high risk of HIV this may have the added benefit of limiting HIV cases.

The guideline provides recommendations on the management of adults with anogenital herpes in the UK. Recommendations include diagnostic tests, management of the primary or first episode of anogenital herpes and recurrences, effectiveness of therapy, prophylaxis, and prevention of transmission between partners as well as patient centred counselling.

### Target users

This guideline has been developed primarily for adults aged 16 years and older presenting to health care professionals working in departments offering Level 3 care in STI management within the United Kingdom. However, the principles of the recommendations should be adopted across all levels; level 1 and 2 providers may need to develop local care pathways where appropriate. Additionally, the principles of the guideline would be relevant to patients of all ages presenting with sexually acquired genital herpes.

### Search strategy

This review was updated by searching PubMed from 2013–2022 for publications in English using the search terms/Mesh headings:

Diagnosis: “Herpes genitalis”, “Herpes simplex diagnosis”.

Neonatal herpes: “Neonatal herpes”, “pregnancy complications – infectious”, “herpes near pregnancy” free text.

A search of the Cochrane Library was also searched using the MeSH terms: “randomized controlled trials”, “Genital Herpes”, “herpes genitalis”.

### Recommendations

Priority was given to randomised controlled trial and systematic review evidence, and recommendations made and graded on the basis of best available evidence using the system published by GRADE system. Conclusions were reached by informal consensus within the writing group.

### Stakeholder involvement and piloting

The current guideline has been developed by the Herpes Simplex Advisory Panel which is a special interest group of the British Association of Sexual Health and HIV (BASHH). The Panel incorporates specialist clinicians, virologists, health advisers, nurses, a clinical psychologist and a representative from the Herpes Viruses Association (a patient support charity).

The draft guideline was placed on the BASHH website for a 2-months consultation period and has been reviewed by the BASHH Public Panel. It was piloted in a representative clinic.

The process was overseen by the Clinical Effectiveness Group of BASHH.

This is the third revision of the UK national guideline first written in 1999.

### Definitions

#### Initial episode

First episode with either herpes simplex virus type 1 (HSV-1) or type 2 (HSV-2). Dependent on whether the individual has had prior exposure to the other type, this is further subdivided into:

##### Primary infection

First infection with either HSV-1 or HSV-2 in an individual with no pre-existing antibodies to either type.

##### Non-primary infection

First infection with either HSV-1 or HSV-2 in an individual with pre-existing antibodies to the other type.

##### Recurrent episode

Recurrence of clinical symptoms due to reactivation of pre-existent HSV-1 or HSV-2 infection after a period of latency.

## Anogenital herpes

### Aetiology

• Herpes simplex virus type 1 (HSV-1, the usual cause of oro-labial herpes and now the most common cause of genital herpes in the UK) or
• Herpes simplex virus type 2 (HSV-2, historically the most common cause of genital herpes in the UK, and the virus type that is more likely to cause recurrent ano-genital symptoms).
• It has been found that 3% of what appear to be typical genital herpes lesions are actually caused by herpes zoster.^1^
• Syphilis may present atypically with multiple tender lesions and therefore be mistaken for herpes simplex.^2,3^
• Mpox virus can also cause papular/blister type eruptions and must now enter the differential diagnosis.

### Natural history

• Only 1/3^rd^ of individuals appear to develop symptoms at the time of acquisition of infection with HSV-2.^4^
• Incubation of infection from acquisition to first clinical signs and symptoms in this minority of individuals ranges from 2 days–2 weeks. In some cases, symptoms can appear years after being infected.^5^
• Infection may be primary or non-primary. Disease episodes may be initial or recurrent and symptomatic or asymptomatic. It is likely that most infections are acquired subclinically.
• Prior infection with HSV-1 modifies the clinical manifestations of first infection by HSV-2, usually making symptoms less severe.^4^
• After childhood, symptomatic primary infection with HSV-1 is equally likely to be acquired in the genital area or oral areas.^6,7^
• Although primary and initial genital herpes in the UK may be caused by HSV-1 or HSV-2, the majority of infections in adults are due to HSV-1. This is more probable in younger age groups (females <50 years, males <35 years).^3^
• Following primary infection, the virus becomes latent in local sensory ganglia, periodically reactivating to cause symptomatic lesions or asymptomatic, but infectious, viral shedding.
• The median recurrence rate for genital herpes after a symptomatic first episode is 0.34 recurrences/month (i.e. approximately four recurrences per year) for HSV-2 and is four times more frequent than the recurrence rate for HSV-1.^8^ Recurrence rates decline over time in most individuals, although this pattern is variable.^9^
• The majority of individuals found to be seropositive for HSV-2 type-specific antibodies subsequently develop symptomatic lesions (once aware of the range of clinical manifestations of HSV-2).^10^ In some of these individuals, the number of days when virus is shed asymptomatically exceeds the number of days of symptomatic shedding associated with lesions. Virus can be shed asymptomatically from the external genitalia, the anorectum, the cervix, and urethra.
• In HIV-positive HSV-2 seropositive individuals, both symptomatic and asymptomatic shedding are increased, especially in those with low CD4 counts and those who are also seropositive for HSV-1.^11,12^

### Clinical features

#### Symptoms

• The patient may be asymptomatic, and the disease unrecognised.
• First symptoms may appear some years after primary infection.^13^
• Local symptoms consist of painful ulceration, dysuria, vaginal or urethral discharge.
• Systemic symptoms are much more common in primary than in non-primary or recurrent disease.
• Systemic symptoms consist of fever and myalgia.
• Rarely, systemic symptoms may be the only evidence of infection.

#### Signs

• Blistering and ulceration of the external genitalia or perianal region (+/− cervix/rectum).
• Tender inguinal lymphadenitis, usually bilateral.
• In first episodes, lesions and lymphadenitis are usually bilateral. In recurrent disease, it is usual for lesions to affect favoured sites. They may alternate between sides but are usually unilateral for each episode. Lymphadenitis occurs in around 30% of patients.
• Recurrent outbreaks are limited to the infected dermatome.

#### Complications

• Superinfection of lesions with candida and streptococcal species (typically occurs in the second week of lesion progression).
• Autonomic neuropathy, resulting in urinary retention.
• Autoinoculation to fingers and adjacent skin e.g. on thighs. Autoinoculation into damaged and inflamed skin has been shown to occur in both acquisition and rarely recurrent disease.
• Aseptic meningitis.

#### Atypical GH

• The lesions of episodes may be small, and may resemble non-specific erythema, erosions or fissures.
• In one study in the US, only approximately 20% of those patients who presented to physicians at a university research clinic with genital symptoms received a correct diagnosis of GH on clinical inspection.^9^ This reflects the fact that a significant proportion of infections due to herpes may present atypically.

#### Herpes proctitis

• HSV is a significant cause of proctitis in MSM. A retrospective review in the USA found that 16% of MSM with proctitis had HSV detected by culture methods, and that 3% had herpes in addition to another rectal STI.^14^
• An Australian study comparing pathogens causing infectious proctitis in HIV positive and HIV negative MSM found HSV proctitis more commonly in HIV positive compared with HIV negative MSM (HSV-1 was found in 14.2% HIV positive and 6.5% HIV negative MSM, and HSV-2 in 22% HIV positive and 12.3% HIV negative MSM). Only 32% of MSM with HSV-associated proctitis had visible external anal ulceration.^15^

### Diagnosis

The diagnostic tests outlined below may not be available in all settings because of local facilities or cost.

#### Virus detection and typing

• The confirmation and typing of the infection, by direct detection of HSV in genital lesions, are essential for diagnosis, prognosis, counselling, and management (1A).
• Methods should be used that directly demonstrate HSV in swabs taken from the base of the anogenital lesion or the rectal mucosa in the case of proctitis. MSM presenting with proctitis should have a rectal swab taken for the detection of HSV (1B).
• Virus typing to differentiate between HSV-1 and HSV-2 should be obtained in all patients with newly diagnosed genital herpes (1B).
• HSV DNA detection by polymerase chain reaction (PCR) increases HSV detection rates by 11–71% compared with virus culture.^16–18^ PCR-based methods allow less stringent conditions for sample storage and transport than virus culture and new real-time PCR assays are rapid and highly specific. Other Nucleic Acid Amplification Test (NAAT) methods have also shown similar results. NAATs are recommended as the preferred diagnostic method for genital herpes (1A). In house PCR assays must be appropriately validated before clinical use. NAATs methods are now regarded as the test of choice. Confirmatory testing of positive PCR samples is currently not considered necessary.
• HSV culture is no longer widely available in diagnostic laboratories, lacks sensitivity, and is not recommended because it misses approximately 30% of PCR positive samples.^16–18^

#### Serology

Key points in interpreting HSV serology:

1. A non type-specific HSV serology test is rarely useful
2. Type specific antibody tests can take time to become positive after infection
3. Patients with proven HSV infection can lose their HSV antibodies
4. HSV IgM testing is rarely useful
5. Serology can be useful for patients e.g.,
  Recurrent genital symptoms and HSV-2 can be considered unlikely in the absence of type-specific antibodies
  Counselling of potentially serodiscordant couples
  In pregnancy when initial herpes is diagnosed in the third trimester

There are two types of HSV antibody tests: type common, which is unable to differentiate between HSV-1 and HSV-2 infections, and type specific, which is able to able to identify previous infection with HSV-1, HSV-2 or both.

Type common antibody tests rely on the high-level of sequence homology between the protein-coding regions of HSV-1 and HSV-2 genomes, resulting in significant antigenic cross reactivity between the two virus types.^19,20^ In contrast, type specific assays are based on the type specific epitopes located on the surface glycoproteins G (gG-1 for HSV-1 and gG-2 for HSV-2), where very limited sequence homology exists.^21–23^ While type common assays can exclude past infection with both HSV types, only type specific antibody tests are clinically useful in the setting of genital herpes.^24,25^

The HSV antibody tests commonly used in clinical practice detect immunoglobulin G (IgG), whose presence indicates infection at some time in the past, without establishing when it occurred. Immunoglobulin M (IgM) assays are not recommended as a marker of recent HSV infections, primarily because they are not type specific and do not confidently differentiate recent from recurrent infections, since an IgM response can be triggered by HSV reactivation.^24,26–28^ In addition, IgM only lasts for 7–14 days, making the test impractical, with venepuncture required within a tight time frame from infection.^26^ HSV-1 and HSV-2 IgG avidity tests, measuring the strength of antigen and antibody binding, which increases over time after the first infection, are not able to differentiate between recent primary infection and past infection. Therefore, primary and non-primary HSV-1 and HSV-2 infections can be only identified by detecting seroconversion to gG-1 or to gG-2 IgG in paired serum samples, collected a few weeks apart, around the time of new genital lesions. However, up to 12% of patients will subsequently lose their IgG antibodies adding yet more complexity to the interpretation of serology.

Western blotting of HSV-1 and -2 infected cell lysates^29–31^ was the first test developed for detecting gG-1 and gG-2 specific antibodies, as well as antibodies common to both virus types and still remains the gold standard test.^32^ However, due to its labour-intensive nature, high cost and complexity in interpretation, it is only suitable as a reference, confirmatory test. Assays for routine clinical use are less labour intensive and most often automated to enable rapid delivery of test results.^33–35^ They utilise a variety of gG-1 and gG-2 antigenic constructs, ranging from whole recombinant proteins to recombinant protein fragments and synthetic peptides. The choice of conserved immunodominant type-specific epitopes is key for ensuring a high sensitivity and specificity to the tests.^36–38^

Commercial assays approved for clinical use are validated comparing their performance against the gold standard method of Western Blot, or against a different immunoassay of known sensitivity and specificity cleared by a regulator (e.g., FDA and CE Mark). The sensitivity of commonly used tests ranges between 91.2 and 100% for HSV-1 and 90.6–100% for HSV-2 while their specificity ranges between 90.1 and 100% for HSV-1 and 95.3 and 100% for HSV-2. There is also considerable time to seroconversion and seroreversion between tests.^33,39^

Seroreversion to anti-IgG seronegative status, sometimes transient, has been documented particularly in individuals with low-level antibody titres and might be related to low antigenic stimulation^27,39^ and in some cases genotypic variation.^37,40–42^

Assay specificity is influenced by antibody cross reactivity, which can occur between some gG-1 and gG-2 epitopes. False positive results are more commonly found associated with low antibody indexes (sample value over cut-off value), typically within three time the cut-off value, as documented with two widely used type specific assays.^32,43,44^

In order to overcome this problem, it has been suggested to raise the cut-off level of the diagnostic test; however, by doing so, the gain in specificity would be associated with a loss in sensitivity. Alternatively, clinical samples with low antibody indexes can be retested using a different assay, ideally with a higher specificity^32,45^(2B). Confirmation of HSV type specific results by a different assay is challenging in the UK, where there is no national reference test available and only a limited number of laboratories offer HSV-1 and HSV-2 type specific antibody assays. In addition, most laboratories utilise the same commercial assay. If a recent seroconversion is suspected, a repeat test on a new serum specimen, collected after at least 3 weeks, identifies the rise in antibody titre after genuine recent infection, acting as confirmation of the initial low-level positive result.

The positive predictive value of a type specific antibody result is affected by the individuals pre-test probability of having contracted the infection and by the prevalence of HSV infection in the tested population. False positive results are more common in low-prevalence populations where the positive predictive value is low (Table 1). Local epidemiological data and patient demographic characteristics should guide testing and result interpretation (2B).^46^

**Table 1.** Positive predictive values for HSV-2 antibody assays.

Clinic location	Prevalence (%)	Positive prediction value^a^
Sexually transmitted infection clinic	25	86%
General population antenatal clinic	5	50%

^a^(for an assay with 95% sensitivity and specificity).

HSV-1 and HSV-2 type specific antibody test results, differently from PCR, do not indicate the site of infection. However, detection of HSV-2 IgG implies anogenital infection, since nearly all HSV-2 infections are acquired sexually; in contrast, detection of HSV-1 IgG does not distinguish between infection acquired through oral-to-oral contact, more typically occurring in childhood, or through sexual activity later in life.^47^

In the USA, the Centre for Disease Control and Prevention does not advise the routine antibody screening of all patients presenting to sexual health clinics. This is because diagnosing genital herpes in someone without symptoms has not shown any change in their sexual behaviour (e.g., wearing a condom or not having sex) nor has it stopped the virus from spreading^32^ There are however situations where HSV antibody testing is beneficial (2B). These include:

— Cases of patients complaining of recurrent genital lesions where HSV PCR is negative. Antibody tests can be useful in ruling out genital herpes in uninfected patients who have symptoms suggestive of HSV infection.
— Counselling patients with initial episode of HSV-1 or HSV-2 anogenital infection confirmed by PCR (to help differentiate recent from established infection), including pregnant women, particularly when the first clinical episode occurs in the third trimester of pregnancy. In this circumstance type specific antibody results inform the mode of delivery, with Caesarean section recommended to women seronegative for the HSV type identified by PCR, due to the significant risk of peripartum transmission to the infant.^27^
— Investigating asymptomatic partners of patients with genital herpes (to identify serodiscordant partners), including couples planning pregnancy, when the male partner has a history of HSV infection and the female partner does not. In this instance HSV type specific serology enables counselling for sexual abstinence in the third trimester of pregnancy for seronegative women and/or advising suppressive antiviral therapy for the male partner.

### Management

#### First episode genital herpes

##### General advice

• Saline bathing
• Analgesia
• Topical anaesthetic agents e.g., 5% lidocaine (lignocaine) ointment may be useful to apply especially prior to micturition. Although the potential for sensitisation exists in the use of topical anaesthetic agents, lidocaine is a rare sensitiser and can be used safely in genital herpes in the form of gel or ointment.^48^

##### Antiviral drugs

• Oral antiviral drugs are indicated within 5 days of the start of the episode, while new lesions are still forming, or if systemic symptoms persist.
• Aciclovir, valaciclovir, and famciclovir all reduce the severity and duration of episodes (1A).^49–51^
• Antiviral therapy does not alter the natural history of the disease in that frequency or severity of subsequent recurrences remains unaltered.^52^
• Topical agents are less effective than oral agents.
• Combining oral and topical treatment is of no additional benefit over oral treatment alone.
• Intravenous therapy is indicated only when the patient cannot swallow or tolerate oral medication because of vomiting.
• There are no comparative studies to show benefit from therapy longer than 5 days or for high dose therapy. However, it may still be prudent to review the patient after 5 days and continue therapy if new lesions are still appearing at this time, or if systemic symptoms are still present, or if complications have occurred. If review is not possible at day 5 a longer course of therapy of 7–10 days is advisable.

##### Recommended regimens (all for 5 days)

• Preferred regimens: aciclovir 400 mg three times daily
  valaciclovir 500 mg twice daily
• Alternative regimens: aciclovir 200 mg five times daily
  famciclovir 250 mg three times daily

##### Management of complications

• Hospitalisation may be required for urinary retention, meningism, and severe constitutional symptoms.
• If catheterisation is required, consideration should be given as to whether a suprapubic approach offers better symptom control to the individual patient.

#### Recurrent Genital Herpes

• Recurrences are self-limiting and generally cause minor symptoms.
• Management decisions should be made in partnership with the patient.
• Strategies include:
   o supportive therapy only
   o episodic antiviral treatments
   o suppressive antiviral therapy.
• The best strategy for managing an individual patient may change over time according to recurrence frequency, symptom severity, and relationship status.

##### General advice (2C)

• Saline bathing
• Petroleum jelly
• Analgesia
• 5% lidocaine ointment

##### Episodic antiviral treatment (1A)

• Oral aciclovir, valaciclovir, and famciclovir reduce the duration and severity of recurrent GH.^53–55^
• The reduction in duration is a median of 1–2 days.
• Head-to-head studies show no advantage of one therapy over another or the advantage of extended 5-days treatment over short course therapy.
• Prodrugs (such as valaciclovir and famciclovir) offer simplified once or twice a day dosing.
• Aborted lesions have been documented in up to a third of patients with early treatment.^56^
• Patient initiated treatment started early in an episode is most likely to be effective, as treatment prior to the development of papules is of greatest benefit.
• Short course therapies offer more convenient and cost-effective strategies for managing GH episodically and should be regarded as first line options.

##### Short course therapies

• Aciclovir 800 mg three times daily for 2 days^57^
• Valaciclovir 500 mg twice daily for 3 days^58,59^
• Famciclovir 1 g twice daily for 1 day^60^

##### Alternative 5-day treatment regimens

• Aciclovir 200 mg five times daily
• Aciclovir 400 mg three times daily for 3–5 days
• Valaciclovir 500 mg twice daily
• Famciclovir 125 mg twice daily

##### Suppressive antiviral therapy

Suppressive antiviral therapy may be considered in a number of situations:

• Control of recurrences
   o Patients who have taken part in trials of suppressive therapy have had to have at least six recurrences per annum. Such patients have fewer or no episodes on suppressive therapy (1A).
   o Patients with lower rates of recurrence will probably also have fewer recurrences with treatment.^61,62^ Suppression therapy may therefore be useful for those with less frequent but painful recurrences which are not adequately controlled with episodic therapy.
• Control of complications
   o Although beyond the scope of these guidelines, antiviral suppression may be useful for the management of those with Mollaret’s meningitis or erythema multiforme.
• Psychosexual problems
  • Patients suffering from psychological morbidity for who the diagnosis causes significant anxiety may benefit from suppressive therapy^63^ which has also been demonstrated to improve quality of life.^64^
  • Reduction of transmission risk: see later section in guidelines.
• Patients should be given full information on the advantages and disadvantages of suppressive therapy. The decision to start suppressive therapy is a subjective one, balancing the frequency of recurrence with the cost and inconvenience of treatment. Patients should be made aware that although suppression tends to reduce recurrence frequency, it may not eliminate them entirely.
• Patient safety and resistance data for long-term suppressive therapy with aciclovir^65^ now extends to over 20 years of continuous surveillance (2B). This confirms that aciclovir is an extremely safe compound requiring no monitoring in previously well patients and only a dose adjustment in those with severe renal disease.

##### Recommended regimens (1A)

• Aciclovir 400 mg twice daily
• Aciclovir 200 mg four times daily^66^
• Valaciclovir 500 mg once daily
• Famciclovir 250 mg twice daily^49^
• Patients should not use once daily aciclovir suppression which has not been shown to be effective and could theoretically increase the risk of the development of antiviral resistance.^66^
  • If breakthrough recurrences occur on standard treatment, the daily dosage should be increased as follows:
     o Aciclovir increased to 400 mg three times daily
     o Valaciclovir increased to 500 mg twice daily
     o Famciclovir increased to 500 mg twice daily.
     o Where higher doses of aciclovir are not effective, consideration may be given to switching to valaciclovir suppression as the decreased dosing frequency may increase adherence. Furthermore, oral bioavailability has been demonstrated to be greater with valaciclovir^67^ although improved suppressive effect over aciclovir has not been demonstrated.
  • Where increased doses of antivirals remain ineffective at controlling recurrences, consideration should be given as to whether symptoms are due to HSV recurrences or whether there is an alternative explanation.
  • Other than routine HIV testing, further immunological investigation of patients with breakthrough recurrences (on suppression) rarely provides any treatable diagnosis.
  • Allergy to aciclovir is rare and, when present, patients may be allergic also to valaciclovir and famciclovir. Famciclovir has been used successfully in some patients with aciclovir allergy, and aciclovir desensitisation has also been performed in some cases. There are no other oral alternatives in use currently for herpes suppression.
  • Choice of treatment depends on patient adherence and cost (Table 2).^140^
  • Patients taking suppressive therapy to reduce the risk of transmission should not have breaks in therapy as transmission risk will increase during such a break. Patients requiring suppressive therapy for psychosexual reasons should not be required to take breaks. For patients taking suppressive therapy to reduce recurrence frequency, they should be advised to discontinue suppression after a maximum of a year to reassess recurrence frequency. The minimum period of assessment should include two recurrences since a recurrence often occurs when ending suppression. Patients who continue to have unacceptably high rates of recurrence or problematic disease may restart treatment. (2C). The risk of aciclovir resistance with long term antiviral use, while well documented in immunosuppressed individuals, has rarely been documented in immunocompetent individuals.
  • Short courses of suppressive therapy may be helpful for some patients (2C). Clinicians need to note that the full suppressive effect is usually only obtained 5 days into treatment.

**Table 2.** Relative costs of antiviral drugs for treating genital herpes.^a^

Indication	Treatment duration	Aciclovir	Famciclovir	Valaciclovir
First episode	5 days	£2.60	£163.35	£2.84
Recurrence	5 days	£1.30	£53.45	£2.84
Suppression	1 year	£33.15	£6380.40	£105.08

^a^Source BNF August 2022. (Different prices may be negotiated by NHS Trusts).

##### Herpes proctitis

• In view of HSV being a common cause of proctitis in MSM, clinicians should consider empirical treatment for HSV in the presence of symptomatic proctitis. Antiviral treatment is as for genital herpes.^68^

### Asymptomatic viral shedding

• Occurs in individuals with genital HSV-1 and those with genital HSV-2.
• Occurs most commonly in patients with genital HSV-2 in the first year after infection and in individuals with frequent symptomatic recurrences.
• Is an important cause of transmission.
• Is reduced by all antiviral therapies.
• For many patients it will decline with time.

### Prevention of Transmission

People with genital HSV should be informed that male condoms, when used consistently and correctly, reduce the risk of genital herpes transmission.^69–72^ Condoms are differentially protective against HSV-2 transmission by sex; condom use reduced per-act risk of transmission from men to women by 96% (*p* < .001) and marginally from women to men by 65% (*p* = .060).

• Aciclovir, famciclovir, and valaciclovir all suppress symptomatic and asymptomatic viral shedding. These drugs have been shown in clinical trials to reduce asymptomatic HSV shedding by about 80–90%. Although the threshold for infection from asymptomatic shedding has not been established, small studies have shown that valaciclovir appears to suppress asymptomatic shedding better than famciclovir.^73^ Aciclovir (400 mg twice daily) has been shown to suppress asymptomatic shedding at least as well as valaciclovir (1000 mg daily).^74^
• Suppressive antiviral therapy with valaciclovir 500 mg once daily reduces the rate of acquisition of HSV-2 infection and clinically symptomatic genital herpes in serodiscordant couples (HR 0.52).^75^ Other antivirals may be effective, but efficacy has not been proven in clinical trials. The valaciclovir transmission study was conducted in monogamous HIV-negative serodiscordant heterosexual couples. The impact of suppressive therapy in preventing transmission in pregnancy, MSM or those that are in HIV serodiscordant relationships should not be presumed where transmissions may occur more easily. Similar studies of HIV-positive patients show limited impact on HSV transmission suggesting that susceptibility is higher or that the levels of suppression of viral shedding is not adequate with currently available antivirals.^76–79^

### Counselling

Discussions regarding HSV should include the natural history, transmission, management of recurrent episodes, episodic and suppressive treatment, disclosure to current and new partners and pregnancy. It is recommended that this should be documented in the notes.

HSV transmission: Key points to cover with patients

• Abstinence from sexual contact is recommended during lesion recurrences or prodromes.
• Transmission may occur as a result of asymptomatic viral shedding. However, the rate of shedding reduces in most people. The low physical morbidity and the high population prevalence should be stressed. A new partner who already has herpes simplex virus is not expected to catch the same type a second time.
• Male condoms, when used consistently and correctly, may reduce the risk of genital herpes transmission, although their use cannot completely prevent it.
• Women are one sixth as likely to infect men as the other way round.
• Persons who are undiagnosed are more apt to transmit infection than those with known infections.
• Transmission does not occur via fomites (sheets, towels, toilets, etc.).
• Suppressive antiviral therapy with antivirals reduces the rate of acquisition of symptomatic genital herpes in serodiscordant couples.
• Telling partners of their infection is recommended in all relationships. Discussions around disclosure and transmission should be documented.

• Diagnosis may cause considerable distress.^80^ Most people with recurrences adjust over time but antiviral treatment can probably reduce anxiety, assist adjustment and improve quality of life. (2D).^63,64,81^
• Care must be taken in all consultations to ensure that appropriate language is used and that alarmist terms (incurable, chronic, attacks) are avoided. Alternative words/phrases are “it could return” and “flare-ups”. Efforts should be made to ensure the patient has understood the information.
• Information and counselling should be as practical as possible and address the patient’s particular situation; issues for someone in a long-term relationship are likely to be different from those for someone with a new or potential new partner.
• Disclosure is often a difficult issue for patients but is more likely to happen in the context of an ongoing relationship. The legal responsibilities and requirements for disclosure remain unclear. Discussions around disclosure should be documented.
• It has been shown that disclosure of the herpes infection to sexual partner may reduce risk of transmission by approximately 50%.^82^
• Everyday stresses do not affect recurrences.
• For most patients one or two counselling sessions with an invitation to return in case of difficulty should be enough.
• Giving all patients a leaflet or contact details for a patient support service such as the Herpes Viruses Association (HVA) may be valuable to an individual patient as much of what has been discussed is forgotten.^83^
• Patients who are still distressed by the diagnosis after a year should be considered for more intensive counselling interventions (2D).
• Information and counselling should cover:
   o Natural history – see section above. The low physical morbidity and high population prevalence should be stressed; It can appear for the first time years after infection^13^; and only one in three people who catch the virus will recognize symptoms and be diagnosed.
   o The use of antiviral drugs for symptom control; current uncertainties about impact on infectivity should be discussed.
   o Discussion of the risks of transmission by sexual contact related to the actual situation of the patient.
   o Reassurance regarding transmission by fomites (sheets, towels, toilets, etc.) and autoinoculation after the first infection is over. However, autoinoculation is a common problem if skin immunity is compromised as occurs in eczema and may be the source of eye infections – usually associated with oral HSV infection.
   o Abstinence from sexual contact during lesional recurrences or prodromes.
   o Transmission may occur as a result of asymptomatic viral shedding.
   o HSV-2 seropositive patients with unrecognised recurrences can be taught to recognise symptomatic episodes after counselling and this may prevent onward transmission.^47-50^
   o The possible benefit of condoms in reducing transmission, emphasizing that their use cannot completely prevent transmission.
   o The re-infection of source partners at genital or distant sites is often a concern. Animal experiments suggest that though possible, the threshold for re-infection is much higher. Currently the evidence for re-infections in humans is limited although some work does suggest this is more likely than previously thought and may be greatest for those who are immunocompromised or have HSV-1.^84^
• Individuals with a current or past history of HSV should understand the importance of not transmitting a new infection to someone who is pregnant. Strategies for avoiding this should be explicitly stated:
   o Conscientious use of condoms during pregnancy, especially from 2 weeks prior to the third trimester as this can reduce transmission to a seronegative pregnant partner. However, condoms may be less effective in preventing transmission than with non-pregnant partners.
   o Abstaining from sex at the time of lesion recurrence and from 2 weeks prior to the third trimester (to term) can also prevent transmission to a seronegative pregnant partner. In addition, this simplifies management of the pregnant patient presenting with an episode of genital ulceration in the third trimester.
   o If the partner has a history of oro-facial HSV, oro-genital transmission to pregnant women should be considered and strategies to avoid transmission discussed.
   o Suppressive antivirals for the partner with infection may also be considered but may be less effective in preventing transmission than with non-pregnant partners.
• Early notification of HSV infection to the midwife and obstetrics team enables discussion and early planning in order to ensure best outcomes for neonates.
• For further detail please see the up to date RCOG and BASHH Herpes in Pregnancy Guidelines.^85^

### Patient support

• The discomfort of symptoms and the stigma associated with HSV infection, as with other conditions,^83^ often results in impaired patient retention of information given by clinical staff.
• See the BASHH Patient Information Leaflet produced in association with this guideline.
• Patients frequently benefit from talking to the Herpes Viruses Association helpline Website: https://www.herpes.org.uk/

### Partner notification

• is not required as a public health measure.
• individuals should be encouraged to disclose their genital herpes status to their sexual partners as a way to decrease transmission.^82^
• is an effective way of detecting individuals with unrecognised disease.^86^
• may clarify whether a partner is infected or not (utilising type-specific antibody testing if necessary – however see ‘Serology’ above about the unreliability of these tests). This may help to relieve anxiety about transmission or reinforce the need to reduce the risk of transmission. Care must be taken in interpreting the result.
• may help with the counselling process.
• Awareness of the diagnosis in a partner or ex-partner may prevent further onward transmission.

### Herpes vaccines

There are no vaccines currently approved for prevention of genital herpes although trials are ongoing. We do not support the use of unauthorized or unlicensed vaccines outside of clinical trials.

### Management of genital herpes in people living with HIV

• There is epidemiological synergy between herpes simplex virus (HSV) and HIV infections.^87,88^ Herpes simplex infections activate HIV replication^89–94^ and may facilitate onward HIV transmission to sexual partners.^95–97^ Suppressive treatment of HSV-2 infection with valaciclovir has been shown to reduce genital HIV shedding in women (not on antiretroviral therapy).^98^ In addition, both prevalent and incident HSV two infections are associated with an increased risk of HIV acquisition.^99,100^
• Genital herpes is a common viral STI in heterosexuals living with HIV in the UK.^101^ The natural history of genital herpes in untreated people living with HIV is significantly different from that in HIV-negative individuals. The most important risk factor for herpes reactivation is the degree of HIV-associated immunosuppression.^102–104^
• Standard systemic antiviral drugs, as used to treat genital herpes in HIV-negative patients, have been shown to successfully treat genital herpes in people living with HIV.^105–110^ Resistance to anti-herpes drugs is more common in those with HIV co-infection and is associated with treatment failure of genital herpes.^111^ Suppressive antiviral therapy with currently available agents has been shown in multiple studies to have no impact on HIV acquisition or transmission risk. HSV treatment used only to manage or reduce HIV transmission or acquisition risk cannot be recommended (1A).^112,113^
• Much of the evidence on herpes management in people living with HIV comes from studies performed before the era of combination antiretroviral therapy; prospective studies performed early in the epidemic showed that clinical lesions might be persistent and progressive in those with HIV. Genital herpes, including chronic erosive lesions may occur as a manifestation of the immune reconstitution inflammatory syndrome (IRIS) following combination antiretroviral therapy.^114–118^ HSV associated IRIS may be unresponsive to previously effective anti-herpes viral therapy in the absence of increased antiviral resistance. Management is difficult but topical cidofovir may be effective.

#### First episode genital herpes

• In the absence of HIV therapy, primary genital herpes may be severe and prolonged with risk of progressive, multifocal and coalescing mucocutaneous anogenital lesions. Moreover, serious and potentially life-threatening systemic complications, such as fulminant hepatitis, pneumonia, neurological disease and disseminated infection have been reported.
• Prompt initiation of therapy is recommended if herpes is suspected clinically. In patients with advanced HIV double the standard dose of antiviral should be considered. If new lesions are still forming after 3–5 days, a repeat viral isolation should be attempted and susceptibility testing arranged if possible (available through Colindale UKHSA laboratory). The dose of HSV therapy should also be increased. Definitive studies in people living with HIV are lacking.
• Recommended regimens^51^
  o Aciclovir 400 mg five times daily for 7–10 days (I, B)
  o Valaciclovir 500 mg-1 gram twice daily for 10 days (I, B)
  o Famciclovir 250–500 mg tid for 10 days (2,C)
• Therapy should be continued until all lesions have re-epithelialized.
• In severe cases, initiation of intravenous therapy with aciclovir 5–10 mg/kg body weight IV every 8 h may be necessary. This should be continued intravenously for 2–7 days, or until clinical improvement, and followed by oral antiviral therapy to complete a minimum of 10 days total treatment (2,D).

#### Recurrent genital herpes

Both clinical and subclinical reactivations of genital herpes are more frequent in people living with HIV and may lead to persistent and progressive anogenital mucocutaneous lesions, especially with CD4 cell counts <50 per mm.^7^ Features can be atypical in nature and hypertrophic lesions can occur. Optimising the control of HIV replication with antiretroviral therapy is of fundamental importance for the management of recurrent genital herpes. ART will reduce the frequency of clinical recurrences but has less effect upon asymptomatic viral shedding. Thereafter, specific antiviral drugs can be used for either episodic or suppressive treatment.

#### Episodic treatment

##### Duration of therapy

• It is likely that 5 days of therapy will be adequate for most patients. It should be noted that in advanced disease many patients will continue to have new lesions developing at the end of a standard 5-days course. Shorter courses of therapy may be adequate in those with higher CD4 counts (>500 cells/mm^3^) although there is very limited trial evidence to support this approach. One trial with famciclovir has reported this effect.^119^

##### Dosage of antivirals

• Providing there is no evidence of immune failure standard doses of antivirals should suffice (1B). In those with advanced disease it may be necessary to double the standard dose and to continue therapy beyond 5 days (1C). Currently there is no evidence to support the use of ultrashort courses of episodic therapy in the immunocompromised.

##### Suppressive treatment

• The efficacy of suppressive antiviral therapy in people living with HIV may be less than in HIV-negative people. It is recommended that intermittent cessation of suppressive antiviral therapy for genital herpes should occur, especially in those in whom there is also sustained HIV viral suppression and rising CD4 cell counts. In some people living with HIV with less frequent outbreaks of genital herpes, episodic treatment may be substituted. In others, where the pre-treatment pattern of recurrences resumes, suppressive treatment may need to restart (2D).
• Recommended drug regimens for daily suppressive treatment^51,120,121^
   o Aciclovir 400 mg orally twice to three times a day
   o Valaciclovir 500 mg orally twice a day
• If these options do not adequately control disease then the first option should be to double the dose. If control is still not achieved, then famciclovir 500 mg orally twice a day can be tried (2C).

##### Impact of HSV suppression on HIV progression

Detectable HIV viraemia has been shown to be reduced with suppressive antiviral therapy with aciclovir or valaciclovir.^122^ This may impact on HIV progression particularly in those patients not on ART. A large RCT in early HIV disease (CD4 counts >250 cells/mm^3^, subjects not on ART) showed that 400 mg bd of aciclovir suppression will sustain CD4 counts above 250 cells/mm^3^ in 16% of subjects compared to placebo. However, benefits of suppressive antivirals have not been demonstrated in the presence of effective antiretroviral treatment.^112,113^

##### Drug resistant genital herpes

• Herpes resistant to aciclovir is very rarely described in the immunocompetent and is usually associated with immunosuppression and not necessarily HIV related^123^
• In prospective studies, aciclovir-resistant strains have been found in around 5%–7% isolates from genital herpes lesions in people living with HIV.^124,125^ Aciclovir resistance is confirmed if isolates require aciclovir concentrations >1–3 mg/l for inhibition.
• Aciclovir resistance is most commonly related to a mutation in the gene encoding HSV thymidine kinase (TK), which is responsible for initial phosphorylation of aciclovir to its active form, resulting in TK that either has reduced affinity for aciclovir or is not synthesised. TK-deficient strains are of reduced pathogenicity in immunocompetent individuals but may cause serious local and systemic disease in severely immunocompromised individuals.^126,127^ They appear less likely to be associated with the development of latency; hence, subsequent clinical reactivations of genital herpes are often caused by aciclovir sensitive isolates. Partially resistant strains may sometimes be successfully treated with high dose intravenous aciclovir and other nucleoside analogues but fully aciclovir-resistant strains are resistant to valaciclovir and ganciclovir, and the majority are resistant to famciclovir.^126–128^ TK-deficient strains are susceptible to foscarnet and cidofovir which do not depend upon TK but which inhibit viral DNA polymerase.
• Antiviral susceptibility testing for HSV is difficult to obtain in the UK but is currently still available through the UKHSA laboratory at Colindale. More often clinical response to antiviral therapy is used to guide decisions, Advice from a clinical virologist about appropriate drug dosages and duration may be sought when clinical resistance is suspected.
• Both topical 1% foscarnet cream^129^ and 1% cidofovir gel^130^ have been shown to produce significant benefits in lesion healing, pain reduction and virological effect in drug resistant herpes in people living with HIV (1A).
• There is limited evidence to support the use of topical trifluorothymidine alone or in combination with interferon-alpha (2D).^131,132^
• Systemic therapy with either foscarnet or cidofovir is generally preferred to treat drug resistant herpes in those living with HIV. There is evidence for foscarnet 40 mg/kg body weight IV every 8 h (I, A)^133,134^ and cidofovir 5 mg/kg body weight weekly IV infusion (2,D)^135–139^ for 2 weeks then every fortnight, administered with oral probenecid and adequate pre-hydration to reduce the risk of nephrotoxicity.
• Alternating courses of treatment with aciclovir and cidofovir for subsequent recurrences has been advocated as a strategy that may reduce the development of cidofovir-resistant strains. The efficacy, safety, and durability of the therapeutic response of these agents has yet to be determined in prospective controlled trials.
• Pritelivir and amenamevir (helicase primase inhibitors) may also be available through compassionate access.^123^

## Auditable outcome measures

• The percentage of cases having attempted herpes simplex virus detection by PCR confirmation. Performance target 97%
• The percentage of cases having at least one detected herpes simplex virus typed. Performance target 97%
• The percentage of cases, presenting within 5 days of the onset of first episode of genital herpes, who were offered recommended antiviral therapy. Performance target 97%
• The percentage of cases, given a diagnosis of genital herpes, who were offered verbal and written information about genital herpes. Performance target 97%.
• The percentage of cases, with six or more herpetic recurrences annually, who were offered recommended suppressive antiviral therapy. Performance target 97%.

The 97% performance standards are to allow for one case in 40 audited not having the recommended documentation owing to a random performance lapse not accounted for in a list of exceptions or exclusions, or a single data entry error.

### Local resource constraints on outcomes

The Guideline authors and the CEG of BASHH do not believe that local resource issues should impact on the delivery of the standards of care as given in this guideline.

### Membership of the CEG

Professor Margaret Kingston

Dr Ade Apoola

Dr Helen Fifer

Dr Sarah Flew

Ms Alison Grant

Dr Deepra Grover

Dr Amy Evans

Dr Nicholas Hardman

Dr Michael Rayment

Dr Ann Sullivan

Dr Suneeta Sonni

### Editorial independence

This guideline was commissioned, edited and endorsed by the BASHH CEG without external funding being sought or obtained.
